# Ultra-processed food consumption by Brazilian adolescents in cafeterias and school meals

**DOI:** 10.1038/s41598-019-43611-x

**Published:** 2019-05-09

**Authors:** Priscilla Rayanne e Silva Noll, Matias Noll, Luiz Carlos de Abreu, Edmund Chada Baracat, Erika Aparecida Silveira, Isabel Cristina Esposito Sorpreso

**Affiliations:** 1 Gynecology Discipline, Department of Obstetrics and Gynecology, Faculdade de Medicina, Universidade de São Paulo (FMUSP), Universidade de São Paulo, São Paulo, Brazil; 2 Instituto Federal Goiano - Campus Ceres, Goiás, Brazil; 3 Faculdade de Medicina do ABC (FMABC), São Paulo, Brazil; 4 Postgraduate Program in Health Sciences, Faculdade de Medicina, Universidade Federal de Goiás (UFG), Goiás, Brazil

**Keywords:** Nutrition, Public health

## Abstract

This cross-sectional study utilized the National School Health Survey 2015 database to assess the association between school cafeterias; the meals offered by the Brazilian School Food Program (PNAE); and the consumption of industrialized/ultra-processed salty foods, sweets, and soft drinks among Brazilian adolescents. A sample of 102,072 adolescents, aged 11–19 years, who were enrolled in the 9th grade completed the survey. The evaluated outcome was the consumption of industrialized/ultra-processed salty foods, sweets, and soft drinks. A Poisson regression model-based multivariate analysis was performed. The effect measure was the prevalence ratio (PR) with its respective 95% confidence intervals (CIs). The results indicated that Brazilian adolescents who attended schools without meals offered through the PNAE had a higher probability of regularly (≥5 times/week) consuming ultra-processed salty foods [PR = 1.06, CI = 1.01–1.11] and soft drinks [PR = 1.08, CI = 1.03–1.14] compared to those who attended schools that offered PNAE meals. Moreover, the presence of a school cafeteria was associated with a higher probability to consume industrialized/ultra-processed salty foods [PR = 1.05, CI = 1.02–1.08], sweets [PR = 1.09, CI = 1.07–1.11], and soft drinks [PR = 1.10, CI = 1.07–1.13]. School meals appear to be associated with the consumption of ultra-processed foods by Brazilian adolescents, indicating areas for health promotion programs.

## Introduction

Being overweight is a global public health problem and a key risk factor for the development of chronic, non-communicable diseases^1–3^. Worldwide, approximately 20–25% of adolescents (individuals aged 10–19 years)^4^ are overweight^1^. In Brazil, 23.7% of adolescents are overweight^5^, which is associated with poor eating habits such as high consumption of ultra-processed foods and low consumption of minimally processed and/or fresh foods^6–10^. Ultra-processed foods are generally defined as those manufactured industrially through several stages of processing^6,7,11^. Accordingly, such foods contain laboratory-synthesized industrial substances (e.g., emulsifiers, colorants, flavorings, flavor enhancers, and thickeners) and large amounts of calories, trans fats, sugars, sodium, and chemical additives^6,7,11^.

For adolescents, school is where messages promoting health, disease prevention, and healthy eating habits can be disseminated^12–14^, including the beneficial effects of healthy meals on learning and school performance^15–19^. However, the presence of a food outlet or cafeteria that sells food either within or near a school is associated with the consumption of foods with low nutrition and high calories, as well as with low vegetable consumption (seen in United States, and the Republic of Ireland)^20,21^. In Australia, for example, there is food with good sources of nutrients; however, it is more expensive than is food that lacks the adequate nutritional value^22,23^. Browne and colleagues^20^ evaluated three different types of school lunches: sourced from home, cafeterias, and take-out from food outlets in the Republic of Ireland. They found that canteens and take-out purchased lunches had worse nutritional value than did packed lunches from home, as well as a greater amount of total fat and added sugars.

Some countries have established policies regarding the sale of foods in schools, which limit foods considered unhealthy (e.g., sweets and soft drinks)^22,24–28^. Positive results were found in France when evaluating the consumption of school canteen lunches when compared with other lunches including the consumption of more fruits, vegetables, fish, and dairy products, and less soft drinks and chocolate^27^. However, in Australia, an evaluation of the policy guidelines to promote nutritious food sales in school canteens revealed unsatisfactory results^22,23^.

In Brazil, school meals are considered a basic human right that plays a key role in student development. Therefore, public schools offer free meals through the Brazilian School Food Program (Programa Nacional de Alimentação Escolar; PNAE), which aims to provide healthy meals for students^29,30^. This comprehensive and long-lasting program has been recognized nationally and internationally by global food and nutrition security organizations^29,30^. Additionally, Brazilian school cafeterias, particularly those in the private school system, also sell food on school premises^5^.

Despite the widespread application of the PNAE in Brazil, studies examining the effects of school meals via the PNAE and school cafeterias on the consumption of ultra-processed foods by adolescents remain scarce, especially at the national level^31^. In this context, it is vital to prevent students from becoming overweight and unhealthy eating habits and identify the influence of food offered by the PNAE and/or sold by school cafeterias on school premises on the consumption of ultra-processed salty foods, soft drinks, and sweets. Therefore, we assessed the association between school cafeterias and the meals offered by the PNAE and the consumption of industrialized/ultra-processed salty foods, sweets, and soft drinks among Brazilian adolescents.

## Results

Overall, 102,072 adolescents were included in the sample and the response rate for all questions was higher than 99.5% for each outcome (industrialized/ultra-processed salty foods, sweets, and soft drinks). The data regarding the consumption of ultra-processed foods, and the results of the bivariate analysis are presented in Tables 1–3.

**Table 1:** Prevalence of the consumption of industrialized/ultra-processed salty foods according to explanatory variables among 9th-grade adolescents in the 2015 PeNSE.

Variable	%	Regular consumption of industrialized/ultra-processed salty foods		
		Prevalence (%)	PR (95% CI)	*p*
**Sociodemographics**				
Municipality (n = 101826)				<0.001
Not capital	49.8	29.2	1	
Capital	50.2	34	**1**.**16 (1**.**13–1**.**19)**	
School (n = 101826)				<0.001
Public	79.5	29	1	
Private	20.5	41.5	**1**.**43 (1**.**40–1**.**47)**	
Sex (n = 101826)				<0.001
Male	48.2	29.7	1	
Female	51.8	33.4	**1**.**13 (1**.**10–1**.**15)**	
Age (n = 101826)				<0.001
11–13 years	16.9	34.4	1	
14 years	50.6	32.3	**0**.**94 (0**.**91–0**.**97)**	
15 years	20.4	30.3	**0**.**88 (0**.**85–0**.**91)**	
16–19 years	12.1	26.8	**0**.**78 (0**.**75–0**.**81)**	
**Availability of food at school**				
PNAE (n = 101651)				<0.001
Yes	82.3	29.5	1	
No	17.7	41.1	**1**.**39 (1**.**36–1**.**43)**	
School cafeteria (n = 101686)				<0.001
No	54.8	28.5	1	
Yes	45.2	35.2	**1**.**23 (1**.**21–1**.**26)**	

PeNSE: Pesquisa Nacional de Saúde do Escolar (National School Health Survey); PNAE: Brazilian School Food Program; PR: prevalence ratio; CI: confidence interval.

**Table 2:** Prevalence of the consumption of sweets according to explanatory variables among 9th-grade adolescents in the 2015 PeNSE.

Variable	%	Regular consumption of sweets		
		Prevalence (%)	PR (95% CI)	*p*
**Sociodemographics**				
Municipality (n = 101890)				0.002
Not capital	49.9	39.1	1	
Capital	50.1	40.3	**1**.**03 (1**.**01–1**.**05)**	
School (n = 101890)				<0.001
Public	79.5	39.3	1	
Private	20.5	41.3	**1**.**05 (1**.**03–1**.**08)**	
Sex (n = 101890)				<0.001
Male	48.3	33.5	1	
Female	51.7	45.5	**1**.**36 (1**.**33–1**.**39)**	
Age (n = 101890)				<0.001
11–13 years	16.9	41.2	1	
14 years	50.6	40.6	**0**.**99 (0**.**96–1**.**01)**	
15 years	20.4	38.6	**0**.**94 (0**.**91–0**.**97)**	
16–19 years	12.1	35.5	**0**.**86 (0**.**83–0**.**89)**	
**Availability of food at school**				
PNAE (n = 101715)				<0.001
Yes	82.3	39.3	1	
No	17.7	41.5	**1**.**06 (1**.**03–1**.**08)**	
School cafeteria (n = 101750)				<0.001
No	54.8	38.1	1	
Yes	45.2	41.6	**1**.**09 (1**.**07–1**.**11)**	

PeNSE: Pesquisa Nacional de Saúde do Escolar (National School Health Survey); PNAE: Brazilian School Food Program; PR: prevalence ratio; CI: confidence interval.

**Table 3:** Prevalence of the consumption of soft drinks according to explanatory variables among 9th-grade adolescents in the 2015 PeNSE.

Variable	%	Regular soft-drink consumption		
		Prevalence (%)	PR (95% CI)	*p*
**Sociodemographics**				
Municipality (n = 101898)				<0.001
Not capital	49.8	24.1	1	
Capital	50.2	27.5	**1**.**14 (1**.**12–1**.**16)**	
School (n = 101898)				0.983
Public	79.5	25.8	1 (0.97–1.03)	
Private	20.5	25.8	1	
Sex (n = 101898)				<0.001
Female	51.7	24.4	1	
Male	48.3	27.4	**1**.**12 (1**.**10–1**.**15)**	
Age (n = 101898)				<0.001
11–13 years	16.9	23.3	1	
14 years	50.6	24.9	**1**.**07 (1**.**04–1**.**10)**	
15 years	20.4	28.5	**1**.**22 (1**.**18–1**.**27)**	
16–19 years	12.1	28.7	**1**.**23 (1**.**19–1**.**28)**	
**Availability of food at school**				
PNAE (n = 101723)				0.073
Yes	82.3	25.7	1	
No	17.7	26.4	1.03 (0.99–1.06)	
School cafeteria (n = 101758)				<0.001
No	54.8	24.9	1	
Yes	45.2	26.9	**1**.**08 (1**.**05–1**.**11)**	

PeNSE: Pesquisa Nacional de Saúde do Escolar (National School Health Survey); PNAE: Brazilian School Food Program; PR: prevalence ratio; CI: confidence interval.

The regular consumption of salty, ultra-processed foods and the consumption of sweets were associated with residing in a capital, private school attendance, female sex, age, absence of PNAE, and presence of a cafeteria (Tables 1 and 2). Regular soft-drink consumption was also associated with residing in a capital, male sex, age, and presence of a cafeteria (Table 3).

Multivariate analyses results are shown in Table 4. Compared to their counterparts, residing in a capital [PR = 1.09, CI = 1.06–1.11], private school attendance, [PR = 1.29, CI = 1.23–1.35], female sex [PR = 1.12, CI = 1.10–1.15], absence of PNAE [PR = 1.06, CI = 1.01–1.11], and presence of a cafeteria [PR = 1.05, CI = 1.02–1.08] were associated with a higher probability of consuming salty foods, while being aged ≥16 years was associated with a lower probability [PR = 0.89, CI = 0.85–0.93]. Second, compared to their counterparts, a higher probability of consuming sweets was associated with female sex [PR = 1.35, CI = 1.33–1.38], and the presence of a cafeteria [PR = 1.09, CI = 1.07–1.11], while being aged ≥16 years was associated with a lower probability [PR = 0.92, CI = 0.88–0.96]. Third, compared to their counterparts, a higher probability of regularly consuming soft drinks was associated with residing in a capital [PR = 1.14, CI = 1.11–1.17], public school attendance [PR = 1.12, CI = 1.06–1.18], male sex [PR = 1.10, CI = 1.08–1.13, increasing age [16–19 years, PR = 1.24, CI = 1.19–1.30], absence of PNAE [PR = 1.08, CI = 1.03–1.14] and the presence of a cafeteria [PR = 1.10, CI = 1.07–1.13].

**Table 4:** Multivariable analyses of factors associated with the consumption of industrialized/ultra-processed salty foods, sweets, and soft drinks by 9th-grade adolescents in the 2015 PeNSE in Brazil.

Variable	Industrialized/ultra-processed salty foods^a^		Sweets^a^		Soft drinks^a^	
	PRa (95% CI)	*p*	PRa (95% CI)	*p*	PRa (95% CI)	*p*
**Sociodemographics**						
Municipality		<0.001		0.144		<0.001
Not capital	1		1		1	
Capital	**1**.**09 (1**.**06–1**.**11)**		1.02 (0.99–1.04)		**1**.**14 (1**.**11–1**.**17)**	
School		<0.001		0.140		<0.001
Public	1		1		**1**.**12 (1**.**06–1**.**18)**	
Private	**1**.**29 (1**.**23–1**.**35)**		0.97 (0.93–1.01)		1	
Sex		<0.001		<0.001		<0.001
Male	1		1		**1**.**10 (1**.**08–1**.**13)**	
Female	**1**.**12 (1**.**10–1**.**15)**		**1**.**35 (1**.**33–1**.**38)**		1	
Age		<0.001		<0.001		<0.001
11–13 years	1		1		1	
14 years	0.98 (0.95–1.01)		1.01 (0.98–1.03)		**1**.**07 (1**.**04–1**.**11)**	
15 years	0.98 (0.94–1.01)		0.98 (0.95–1.02)		**1**.**23 (1**.**18–1**.**28)**	
16–19 years	**0**.**89 (0**.**85–0**.**93)**		**0**.**92 (0**.**88–0**.**96)**		**1**.**24 (1**.**19–1**.**30)**	
**Availability of food at school**						
PNAE		0.014		0.463		0.003
Yes	1		1		1	
No	**1**.**06 (1**.**01–1**.**11)**		1.02 (0.97–1.06)		**1**.**08 (1**.**03–1**.**14)**	
School cafeteria		<0.001		<0.001		<0.001
No	1		1		1	
Yes	**1**.**05 (1**.**02–1**.**08)**		**1**.**09 (1**.**07–1**.**11)**		**1**.**10 (1**.**07–1**.**13)**	

The multivariable analysis was conducted using the Poisson regression model. The effect measure is the PRa with its respective 95% CI. The model was adjusted for all explanatory variables.

PeNSE: Pesquisa Nacional de Saúde do Escolar (National School Health Survey); PNAE: Brazilian School Food Program; PRa: adjusted prevalence ratio; CI: confidence interval.

Bolded p-values denote significance (*p* < 0.05).

^a^Regular consumption (at least 5 of the previous 7 days).

## Discussion

This study assessed the association between school cafeterias; the meals offered by the PNAE; and the consumption of industrialized/ultra-processed salty foods, sweets, and soft drinks among Brazilian adolescents. The results indicate that 9th graders who attended schools covered by the PNAE have a lower probability of consuming ultra-processed salty foods and soft drinks, whereas the probability to consume ultra-processed salty foods, sweets, and soft drinks was higher for those who attend schools with cafeterias. Given the paucity of research related to the influence of meals offered by the PNAE and school cafeterias on the consumption of ultra-processed food by adolescents, our study represents a significant contribution to this field.

Students attending schools covered by the PNAE reported a lower consumption of salty, ultra-processed foods and soft drinks. This is consistent with previous findings that national food programs, including the PNAE, tend to prioritize the provision of a healthy diet, defined as a greater quantity of fresh or minimally processed foods such as fruits, vegetables, and milk, and reduced levels of sodium and simple sugars^17–19,32–34^. In the Philippines, a school meal program developed nutritious meals for adolescents to ensure higher intakes of micronutrients (*p* < 0.001), fiber (*p* < 0.001), and the intake of higher-quality fats (*p* < 0.001) as compared with basal meals^35^. In France, school meals were associated with the consumption of fresh vegetables and fruits (*p* < 0.001) and family income (*p* < 0.001). Specifically, the diet provided in France increased the intake of vegetables and fruits among adolescents with a low socioeconomic status, which reduced the differences between them and their peers with a higher socioeconomic status^34^.

In Brazil, the evaluation of school meals through the PNAE in public schools remains challenging because adherence rates are relatively low (38–62%) and institutions tend not to follow the program guidelines strictly^31,36,37^. Despite these limitations, offering school meals via the PNAE is associated with an increased consumption of fresh foods and a lower consumption of ultra-processed foods^31,38^. This may be attributable to PNAE restrictions on the use of ultra-processed foods, such as processed meats, canned goods, sugary beverages, and concentrated and/or prepackaged foods^32,39^.

By contrast, the presence of a school cafeteria was associated with an increased probability that students would regularly consume industrialized/ultra-processed salty foods, sweets, and soft drinks in this study. Although school meals encompass all food offered in the school environment, regardless of origin, the school cafeteria is often decontextualized in relation to healthy eating^18,23,25,28,40–42^. Despite policies related to healthy food supply from school cafeterias in several countries, the implementation and enforcement of such policies remains limited, even in developed countries such as the United States, New Zealand, United Kingdom, and Australia^18,23,25,28,41,42^. In Australia, for example, even with the implementation of policy guidelines for the promotion of healthy food sales in 2005, an evaluation of government school menus found that most school cafeterias (especially in unmonitored schools) did not comply with the guidelines and continued to offer prohibited foods such as sweets and soft drinks^28^. In Brazil, no national law currently prohibits or restricts the sale of ultra-processed foods within the school environment, despite the PNAE legislation defining school meals as any food offered in the school environment. Nevertheless, there are laws prohibiting the sale of certain foods, especially sweets and soft drinks in some states (e.g., São Paulo, Santa Catarina, Amazonas, and Goiás) and municipalities (e.g., Florianópolis and Rio de Janeiro)^32,39^.

Interventional studies aiming to change the profile of available foods in school cafeterias by reducing the availability of ultra-processed and processed foods and increasing that of fresh or minimally processed foods and/or meals have yielded positive results concerning improved nutritional profiles and adherence to a healthy diet among adolescents^23,35,41,43,44^. These changes in available foods yielded net reductions in the caloric, sugar, and sodium contents of the offered meals^41^. However, the significant cost difference between a healthy diet and ultra-processed foods must be considered^22,44^.

We found that adolescents enrolled in private schools had a greater probability of regularly consuming ultra-processed salty foods compared to those in public schools. This may be attributable to the presence of PNAE-provided school foods in the latter, which protects against the regular consumption of ultra-processed salty foods^31,45^. Similarly, students who attended schools in capital cities had a greater probability of regularly consuming ultra-processed salty foods and soft drinks than did students who attended other schools, likely because of the greater availability of food outlets or cafeterias inside or near the schools^36,44,46^. However, public school students had a greater probability of regularly consuming soft drinks. This may be due to the fact that PNAE-provided school foods do not exclude the consumption of soft drinks when adolescents have access to these beverages in the school environment^47^. Moreover, a study from 124 Brazilian municipalities noted that there are food outlets or cafeterias inside or near the schools in 58.6% and 47.7% of public and private schools, respectively^45^.

Additional barriers to the initial and adequate implementation of existing policies and/or policy making in countries that have not yet legislated school meals are related to inadequate resources, limited legislation regarding consumption quantities, lack of knowledge about healthy foods, and costs^44,48^. Public policies must be continuously updated, implemented, and monitored to ensure a healthy food environment that will promote good health and appropriate physical, psychological, and social development of adolescents.

The main limitation of this study was its cross-sectional design; thus, we cannot infer causality. Moreover, using a restricted sample composed of 9th-grade students, who may not be inclined to accept foods offered at school, was a limitation. Additionally, we cannot state that these data reflect adolescents’ usual food consumption since the information was self-reported; therefore, over- and under-estimation are both possible. Lastly, some significant results should be interpreted with caution because of the large sample size.

In conclusion, school meals appear to be associated with the consumption of ultra-processed foods by Brazilian adolescents. Specifically, the provision of school meals via the PNAE was associated with less consumption of industrialized/ultra-processed salty foods and soft drinks, whereas the presence of a school cafeteria was related to a higher consumption of industrialized/ultra-processed salty foods, sweets, and soft drinks. These findings address an existing gap in the literature and provide data indicating areas for potential improvement.

Our results inform intervention strategies to promote healthy eating patterns and prevent the development of chronic, non-transmissible diseases among adolescents and young adults. The consumption of ultra-processed food is associated with several health concerns obesity, cardiovascular diseases, diabetes, and cancer^3,10,49,50^, compromising individuals’ quality of life during adolescence and later in adulthood. Therefore, schools have a crucial role in promoting students’ healthy eating^51^.

## Methods

### Data sources and study population

This cross-sectional study was conducted using the National School Health Survey (Pesquisa Nacional de Saúde do Escolar; PeNSE) 2015 database^52^, through a partnership between the Ministry of Health and the Brazilian Institute of Geography and Statistics (Instituto Brasileiro de Geografa e Estatística; IBGE). The study results were made available by the IBGE in 2016^53^. The PeNSE was approved by the National Commission on Ethics in Research (Comissão Nacional de Ética em Pesquisa; CONEP) of the National Health Council, which regulates and approves health research involving human participants (CONEP resolution no. 1,006,467; March 30, 2015).

This study involved adolescents aged 11–19 years who were classified as 9th graders in both public and private schools throughout Brazil from April to September 2015. This sample of adolescents adequately represents Brazil, including the 27 federative units (26 states with its capitals and municipalities, and the Federal District; IBGE)^53^.

The sample size allowed for an estimation of the parameters for each of the 26 states and the Federal District, including capitals and municipalities from the interior of Brazil. Brazil is conventionally divided into five regions (North, Northeast, Southeast, South, and Midwest)^53^.

The samples of the geographic levels comprising capitals and municipalities were random and equiprobabilistic; they were calculated using the following parameters: 0.03% maximum error, 95% confidence level, and prevalence of 0.5. Overall, 120,122 students who were enrolled in and attended one of 4,159 classes across 3,040 schools were included in the 2015 sample. Of these, 102,072 students completed the survey on the sampling day. As all students in the sampled classes were invited to respond to the survey questionnaire, there was a sample loss of approximately 15%^53^.

All students that agreed to participate voluntarily provided written, informed consent. Students were told that they could leave the study at any time if they chose not to participate in any of the procedures^53^. Additionally, this study was verified in accordance with the Strengthening the Reporting of Observational Studies in Epidemiology (STROBE) checklist^54^.

### Outcome measures

The PNAE is a national program based on a public food policy^29,30^. Accordingly, this program provides free meals to all basic education students in the public education network (municipal, state, and federal) and covers 200 school days per year. The PNAE aims to foster students’ growth and development, encourage the formation of healthy eating habits, and provide food and nutrition education^29,32^. As noted in the Introduction, school cafeterias also sell food on the premises of educational institutions, especially private schools.

Data collection, involving a validated self-administered survey^55^, was performed using smartphones distributed to the students who were in class on the day of the interview by the IBGE technician. The technician explained to students how to use the device to complete the questionnaire^53^. We considered the consumption of ultra-processed food as an outcome for which three different categories were set according to food and/or food groups: industrialized/ultra-processed salty processed foods (salty, canned, and instant foods), sweets (sweets, candies, chocolate, chewing gum, and lollipops), and soft drinks.

These variables were evaluated using the following question: “In the last 7 days, on how many days did you eat/drink…?” The eight available response options were “I did not eat/drink in the last 7 days”, “I ate/drank on 1 (2, …, 6) of the last 7 days”, and “I ate/drank on all of the last 7 days”. For data analysis purposes, the food groups were categorized as regularly consumed (at least 5 of the previous 7 days) or not regularly consumed^37,56–58^.

### Statistical analyses

Data were analyzed using descriptive statistics and the Wald chi-square test of association (bivariate analysis) for the following outcome groups: industrialized/ultra-processed salty foods, sweets, and soft drinks. The following factors were considered explanatory group variables: sex, age, municipality type, school type, availability of food offered by the PNAE, and presence of a school cafeteria. All variables are included in Poisson regression model-based multivariate analysis and the effect measured was the prevalence ratio (PR) with the respective 95% confidence intervals (CIs)^59^. Methodological studies support the inclusion of variables with theoretical grounds in the final multivariable statistical analyses^60,61^. Statistical analyses were performed using SPSS 20.0 (IBM, Armonk, NY, USA).

## Supplementary information

Dataset 1

## References

[1] Ng M (2014). Global, regional, and national prevalence of overweight and obesity in children and adults during 1980–2013: a systematic analysis for the Global Burden of Disease Study 2013. Lancet.

[2] Ogden CL (2016). Trends in Obesity Prevalence Among Children and Adolescents in the United States, 1988–1994 Through 2013–2014. Jama.

[3] World Health Organization. *Global health risks: mortality and burden of disease attributable to selected major risks*. (WHO Press, 2009).

[4] World Health Organization. Adolescent health. Available at, https://www.who.int/topics/adolescent_health/en/. (Accessed: 19th December 2018). (2018).

[5] Ministério da Saúde & IBGE. *Pesquisa Nacional de Saúde do Escolar 2015*. *Instituto Brasileiro de Geografa e Estatística* (2016).

[6] Moubarac J (2013). International differences in cost and consumption of ready-to-consume food and drink products: United Kingdom and Brazil, 2008–2009. *Glob*. Public Health.

[7] Monteiro CA, Cannon G (2012). The impact of transnational “big food” companies on the south: a view from Brazil. PLoS Med..

[8] Swinburn B (2013). Monitoring and benchmarking government policies and actions to improve the healthiness of food environments: a proposed Government Healthy Food Environment Policy Index. Obes. Rev..

[9] Fortin B, Yazbeck M (2015). Peer effects, fast food consumption and adolescent weight gain. J. Health Econ..

[10] World Health Organisation (2003). FAO/WHO Expert Consultation. Diet, nutrition and the prevention of chronic diseases: report of a joint WHO/FAO expert consultation, Geneva.

[11] Fardet A (2015). Current Food Classifications in Epidemiological Studies Do Not Enable Solid Nutritional Recommendations for Preventing Diet-Related Chronic Diseases: The Impact of Food Processing. Adv. Nutr. An Int. Rev. J..

[12] Wechsler, H., Devereaux, R. S., Davis, M. & Collins, J. Using the school environment to promote physical activity and healthy eating. *Prev*. *Med*. *(Baltim)*. **31** (2000).

[13] European Union. EU Action Plan on Childhood Obesity 2014–2020 (2014).

[14] World Health Organization (WHO). Report of the Commission on Ending Childhood Obesity. *World Health Organization* (2016).

[15] Mozaffarian D (2015). Executive Summary: Heart Disease and Stroke Statistics–2015 Update: A Report From the American Heart Association. Circulation.

[16] ISHIDA H (2015). Role of School Meal Service in Nutrition. J. Nutr. Sci. Vitaminol. (Tokyo)..

[17] Hirschman J, Chriqui JF (2013). School food and nutrition policy, monitoring and evaluation in the USA. Public Health Nutr..

[18] Schwartz MB, Henderson KE, Read M, Danna N, Ickovics JR (2015). New School Meal Regulations Increase Fruit Consumption and Do Not Increase Total Plate Waste. Child. Obes..

[19] Ohri-Vachaspati P (2014). Parental perception of the nutritional quality of school meals and its association with students’ school lunch participation. Appetite.

[20] Browne S (2017). School lunches in the Republic of Ireland: A comparison of the nutritional quality of adolescents’ lunches sourced from home or purchased at school or ‘out’ at local food outlets. Public Health Nutr..

[21] Poti JM, Slining MM, Popkin BM (2014). Where are kids getting their empty calories? Stores, schools, and fast-food restaurants each played an important role in empty calorie intake among US children during 2009–2010. J. Acad. Nutr. Diet..

[22] Wyse R (2017). The price of healthy and unhealthy foods in Australian primary school canteens. Aust. N. Z. J. Public Health.

[23] Yoong SL (2016). CAFÉ: A multicomponent audit and feedback intervention to improve implementation of healthy food policy in primary school canteens: A randomised controlled trial. Int. J. Behav. Nutr. Phys. Act..

[24] Bertin M, Lafay L, Calamassi-Tran G, Volatier JL, Dubuisson C (2012). School meals in French secondary state schools: Do national recommendations lead to healthier nutrition on offer?. Br. J. Nutr..

[25] Adamson A (2013). School food standards in the UK: Implementation and evaluation. Public Health Nutr..

[26] Reilly K (2016). Validity of four measures in assessing school canteen menu compliance with state-based healthy canteen policy. Heal. Promot. J. Aust..

[27] Dubuisson C (2015). The relationship between school lunch attendance and the food intakes of French schoolchildren aged 3–17 years. Public Health Nutr..

[28] Woods J, Bressan A, Langelaan C, Mallon A, Palermo C (2014). Australian school canteens: Menu guideline adherence or avoidance?. Heal. Promot. J. Aust..

[29] Peixinho AML (2013). The trajectory of the Brazilian School Nutrition Program between 2003 and 2010: report of the national manager. Cien. Saude Colet..

[30] Food and Agriculture Organization of the United Nations. Strengthening school feeding programs in the framework of the Zero Hunger Initiative in Latin America and the Caribbean 2025 (2015).

[31] Master NTLT, Canella DS, Bandoni DH (2018). Positive influence of school meals on food consumption in Brazil. Nutrition.

[32] BRASIL. Lei n° 11.947, de 16 de junho de 2009 (2012).

[33] WOO T (2015). The School Meal System and School-Based Nutrition Education in Korea. J. Nutr. Sci. Vitaminol. (Tokyo)..

[34] Longacre MR (2014). School food reduces household income disparities in adolescents’ frequency of fruit and vegetable intake. Prev. Med. (Baltim)..

[35] Angeles-Agdeppa I (2014). Energy and nutrient intake and acceptability of nutritionally balanced school meals in Filipino students. Food Nutr. Bull..

[36] Locatelli, N. T., Canella, D. S. & Bandoni, D. H. Factors associated with the consumption of school meals by Brazilian adolescents: results of the PeNSE survey 2012. *Cad*. *Saude Publica* **33** (2017). 10.1590/0102-311X00183615 28538796

[37] Azeredo CM (2014). Dietary intake of Brazilian adolescents. Public Health Nutr..

[38] Bento Bruna M.A., Moreira Andressa da C., Carmo Ariene S. do, Santos Luana C. dos, Horta Paula M. (2018). A higher number of school meals is associated with a less-processed diet. Jornal de Pediatria.

[39] Brasil. Resolução CD/FNDE n° 26, de 17 de junho de 2013. 1–44 (2013).

[40] Nh NR, Manan W, Muda W, Izani N, Jamil N (2017). How Healthy Is Competitive Food Served at Primary School Canteen in Malaysia?. Int. Med. J..

[41] Cummings PL (2014). Nutrient content of school meals before and after implementation of nutrition recommendations in five school districts across two U.S. counties. Prev. Med. (Baltim)..

[42] Wolfenden L (2014). A randomised controlled trial of an intervention to increase the implementation of a healthy canteen policy in Australian primary schools: Study protocol. Implement. Sci..

[43] Rajbhandari-Thapa J (2017). Effect of the Strong4Life School Nutrition Program on Cafeterias and on Manager and Staff Member Knowledge and Practice, Georgia, 2015. Public Health Rep..

[44] Reilly, K. L. *et al*. Economic analysis of three interventions of different intensity in improving school implementation of a government healthy canteen policy in Australia: costs, incremental and relative cost effectiveness. 1–9 (2018). 10.1186/s12889-018-5315-y PMC5859495 29558931

[45] Carmo ASD, Assis MMD, Cunha CDF, Oliveira TRPRD, Mendes LL (2018). The food environment of Brazilian public and private schools. Cad. Saude Publica.

[46] Virtanen Marianna, Kivimäki Hanne, Ervasti Jenni, Oksanen Tuula, Pentti Jaana, Kouvonen Anne, Halonen Jaana I., Kivimäki Mika, Vahtera Jussi (2015). Fast-food outlets and grocery stores near school and adolescents’ eating habits and overweight in Finland. The European Journal of Public Health.

[47] Datar A, Nicosia N (2017). The Effect of State Competitive Food and Beverage Regulations on Childhood Overweight and Obesity. J. Adolesc. Heal..

[48] Barbosa NVS, Machado NMV, Soares MCV, Pinto ARR (2013). School nutrition and autonomy - challenges and opportunities. Cien. Saude Colet..

[49] Ruiz-Núñez B, Pruimboom L, Dijck-Brouwer DAJ, Muskiet FAJ (2013). Lifestyle and nutritional imbalances associated with Western diseases: causes and consequences of chronic systemic low-grade inflammation in an evolutionary context. J. Nutr. Biochem..

[50] World Health Organization. Young people’s health - a challenge for society. 120 (1986).

[51] Catalano RF (2012). Worldwide application of prevention science in adolescent health. Lancet.

[52] Intituto Brasileiro de Geografia e Estatística. Pesquisa Nacional de Saúde do Escolar - PeNSE. (2017).

[53] Oliveira MMde (2017). Characteristics of the National Adolescent School-based Health Survey – PeNSE, Brazil. Epidemiol. e Serviços Saúde.

[54] Elm Evon (2007). Strengthening the reporting of observational studies in epidemiology (STROBE) statement: guidelines for reporting observational studies. BMJ.

[55] Tavares LF (2014). Validade relativa de indicadores de práticas alimentares da Pesquisa Nacional de Saúde do Escolar entre adolescentes do Rio de Janeiro, Brasil. Cad. Saude Publica.

[56] Machado C (2016). Food environments in schools and in the immediate vicinity are associated with unhealthy food consumption among Brazilian adolescents. Prev. Med. (Baltim)..

[57] Mathur C, Stigler M, Lust K, Laska M (2014). A Latent Class Analysis of Weight-Related Health Behaviors Among 2- and 4-Year College Students and Associated Risk of Obesity. Heal. Educ. Behav..

[58] Christofaro DGD, De Andrade SM, Mesas AE, Fernandes RA, Farias Júnior JC (2016). Higher screen time is associated with overweight, poor dietary habits and physical inactivity in Brazilian adolescents, mainly among girls. Eur. J. Sport Sci..

[59] Barros AJ, Hirakata VN (2003). Alternatives for logistic regression in cross-sectional studies: an empirical comparison of models that directly estimate the prevalence ratio. BMC Med. Res. Methodol..

[60] Sun GW, Shook TL, Kay G (1996). Inappropriate use of bivariable analysis to screen risk factors for use in multivariable analysis. J. Clin. Epidemiol..

[61] Harrell, F. E. *Regression Modeling Strategies: With Applications to Linear Models*, *Logistic Regression and Survival Analysis*. (Springer-Verlag, 2001).

